# Brain Metastases as Presenting Feature in 'Burned Out' Testicular Germ Cell Tumor

**DOI:** 10.7759/cureus.551

**Published:** 2016-04-01

**Authors:** Kate Johnson, Bryan Brunet

**Affiliations:** 1 Radiation Oncology, Cancer Care Manitoba, University of Manitoba, Canada; 2 Radiation Oncology, Saskatoon Cancer Center

**Keywords:** brain metastasis, regressed, 'burned out' phenomenon, testicular tumour, germ cell tumour, chromosome 12p, case report

## Abstract

Testicular germ cell tumors (TGCTs) are the most common malignancy in males aged 20 to 39, and the incidence is increasing. TGCTs have a tendency to grow rapidly with a high risk of metastatic spread. TGCTs generally present with a palpable testicular mass, yet may present less commonly with symptoms arising from metastatic disease.

A 24-year-old otherwise healthy male presented with progressive headaches. Initial imaging reported a single mass in the right frontal lobe. Complete surgical resection revealed suspicion for metastatic poorly differentiated carcinoma with an inconclusive immunohistochemical profile. Further staging scans revealed pulmonary and pelvic tumor deposits. Tumor markers with alpha-fetoprotein, beta-human chorionic gonadotropin, and lactate dehydrogenase were not elevated. Follow-up cranial magnetic resonance imaging revealed intracranial disease progression and he underwent whole brain radiation therapy. Additional outside pathology consultation for chromosomal analysis revealed features consistent with a TGCT. A scrotal ultrasound revealed a minimally atrophic right testicle. With evidence supporting the potential for response to chemotherapeutic treatment in TGCT, the patient was started on cisplatin and etoposide. Bleomycin was planned for the second cycle of chemotherapy if his pulmonary function improved.

A salient feature of all invasive TGCTs is a gain in material in the short arm of chromosome 12, and is diagnostic if present. Although the initial pathology revealed a non-diagnostic metastatic tumor, further testing revealed amplification of chromosome 12p. The examination of poorly differentiated carcinomas of an unknown primary site using light microscopy and immunohistochemical profiling alone may be inadequate, and should undergo molecular chromosomal analysis.

This case is presented for its unconventional presentation and rarity of occurrence. It brings forward the discussion of both the commonality of TGCT in young male adults, as well as the anomaly of a 'burned out' phenomenon. With unreliable tumor markers, nonspecific symptoms, and pathological findings, ‘burned out’ TGCTs may account for a challenging diagnosis in a variety of cases, especially with the presenting symptom arising from a less common metastatic site. This case adds to the increasing literature on a rare entity of the 'burned out' TGCT, and upon literature review, presents itself as the first reported case presenting with brain metastasis.

## Introduction

Testicular germ cell tumors (TGCTs) are the most common malignancy diagnosed in males aged 20 to 39, and the incidence is increasing [1-3]. TGCTs have a tendency to grow rapidly with a high risk of metastatic spread. TGCTs generally present with a palpable testicular mass, yet, less commonly may present with symptoms arising from metastatic disease. Specifically, TGCTs have a propensity to metastasize to retroperitoneal lymph nodes, lungs, liver, bones, and less frequently, to the brain [4].

The phenomenon of a primary TGCT outgrowing its blood supply and undergoing auto-infarction has been described as a ‘burned out’ TGCT. The regressed testicular lesion is not appreciable on physical exam, and spontaneous regression occurs without treatment [5]. Despite the regression of the primary testicular tumor, approximately 50% of ‘burned out’ primary testicular tumors continue to harbor malignant cells and distant metastatic disease can progress [6-7]. A ‘burned out’ TGCT can arise, regress, and metastasize within the same testicle. Cases within the literature describe pathological evidence of tumor regression of a testicular mass with a focus of GCT within a clinically unremarkable testicle [4,8]. This can lead to difficulty in making a diagnosis as the metastasis can be mistaken for a primary tumor.

Imaging can be helpful in making the diagnosis, with scrotal ultrasonography revealing evidence of a regressed tumor. Possible findings consist of a hypoechoic area, atrophic testicle, or microcalcifications [7-8]. Macroscopic evidence of a fibrotic scar in the parenchyma and microscopic findings of intratubular germ cells or seminomatous foci may be seen on pathological evaluation [9-11].

Of significance, extra-gonadal germ cell tumors (EGCT) are a known entity that also present with biochemistry and histological findings of a germ cell tumor in the absence of primary testicular or ovarian tumor. However, EGCT are differentiated from ‘burned out’ TGCT by their characteristic midline location, from the pineal gland to the coccyx. Furthermore, in EGCT no radiologic nor pathologic evidence of a primary malignancy is present in the primary reproductive organs [12].

Chemotherapeutic strategies implemented in the 1970s for the treatment of advanced stage TGCTs represents a paradigm shift to a curable disease [13-15]. Here we discuss a rare case that highlights the challenges of diagnosing a ‘burned out’ TGCT.

## Case presentation

**Figure 1:**
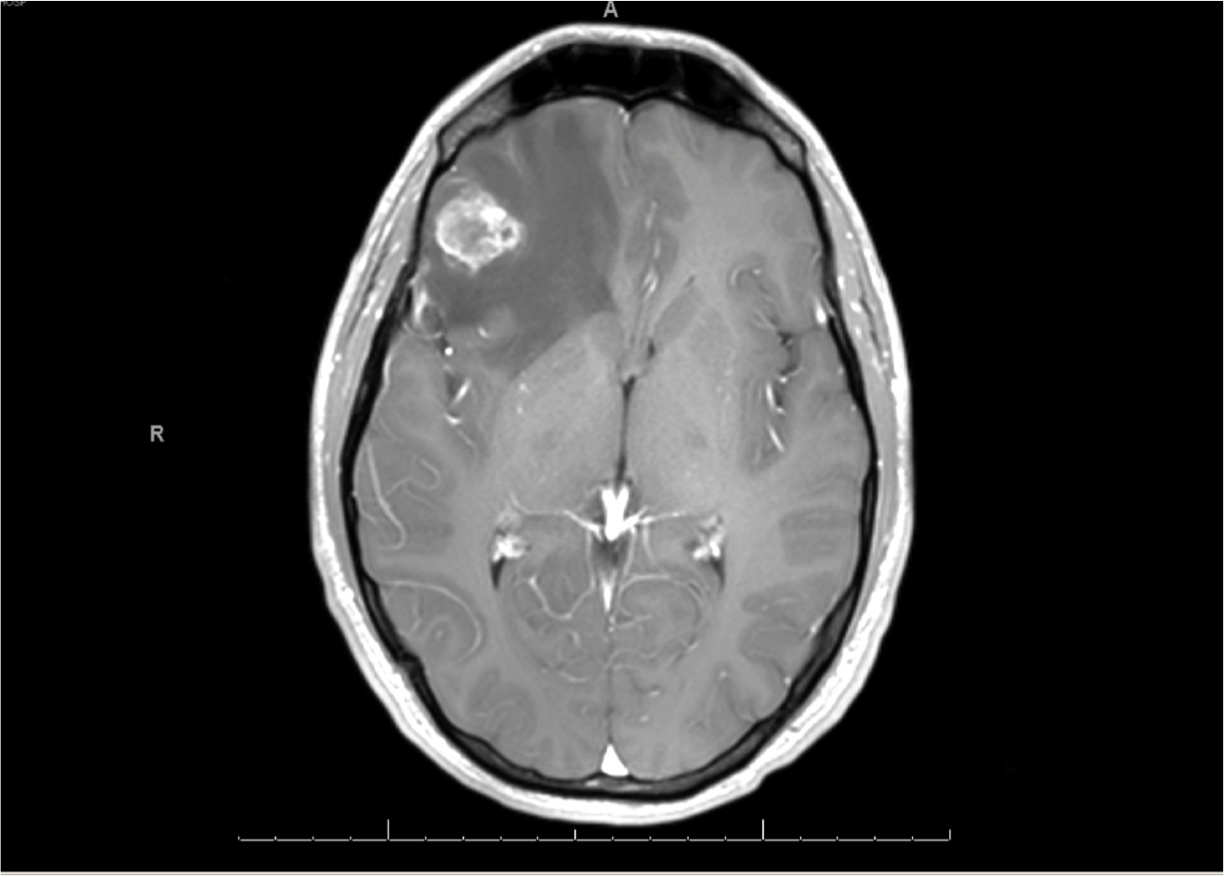
Initial Brain MRI Single intra-axial heterogeneously enhancing mass in the inferior aspect of the right frontal lobe.

A 24-year-old previously healthy male presented with progressive nausea, vomiting, visual changes, and memory impairment. His only significant finding on history was a strong family history of factor V Leiden mutation. The physical exam was grossly unremarkable. The initial magnetic resonance imaging (MRI) reported a single mass in the right frontal lobe (Figure 1).

With high suspicion for primary brain tumor, total resection of the intracranial lesion was performed and revealed a metastatic, poorly differentiated carcinoma with an inconclusive immunohistochemical profile.

**Figure 2:**
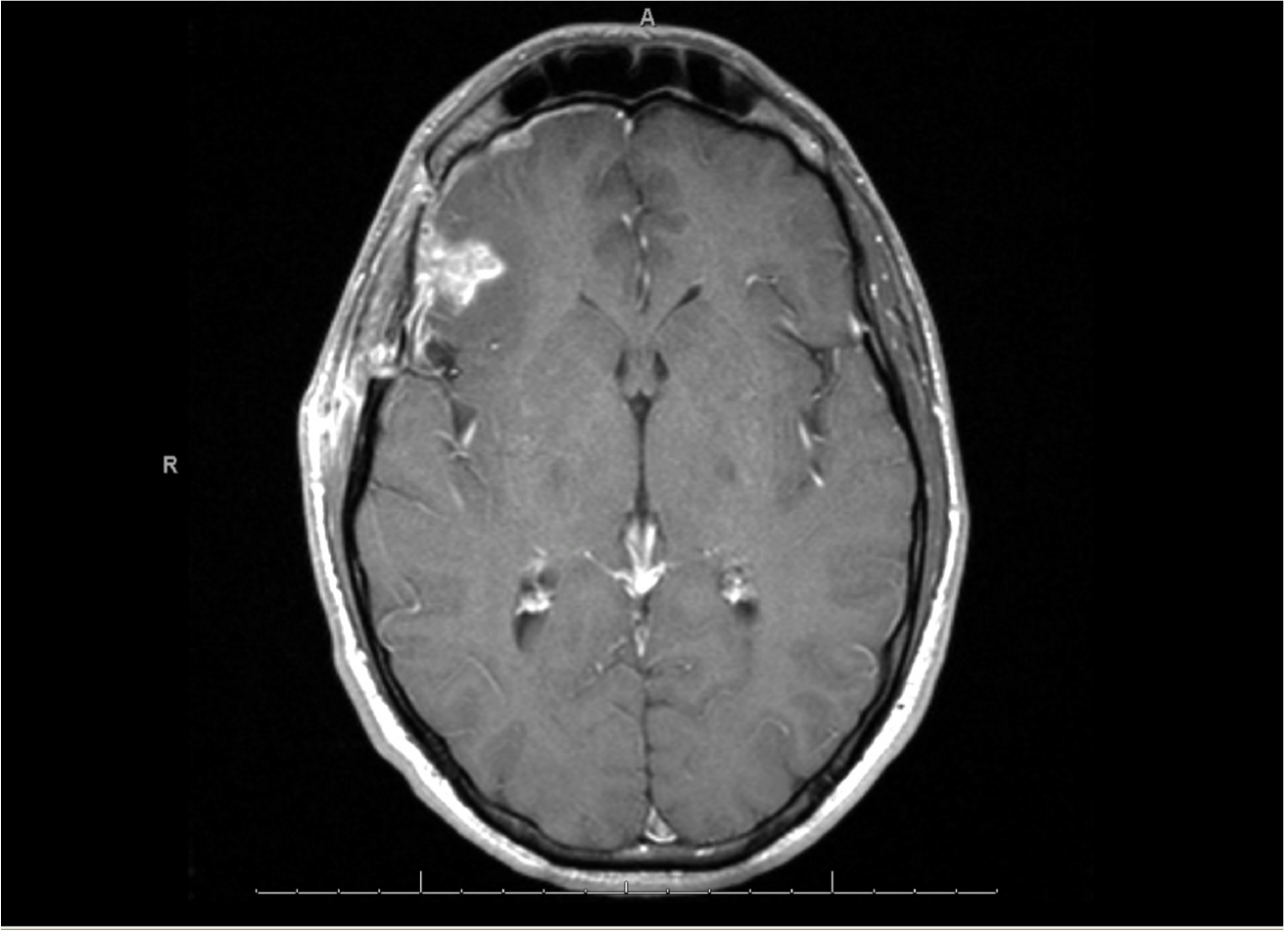
MRI One Month Post-Resection Increased enhancement in the surgical bed.

**Figure 3:**
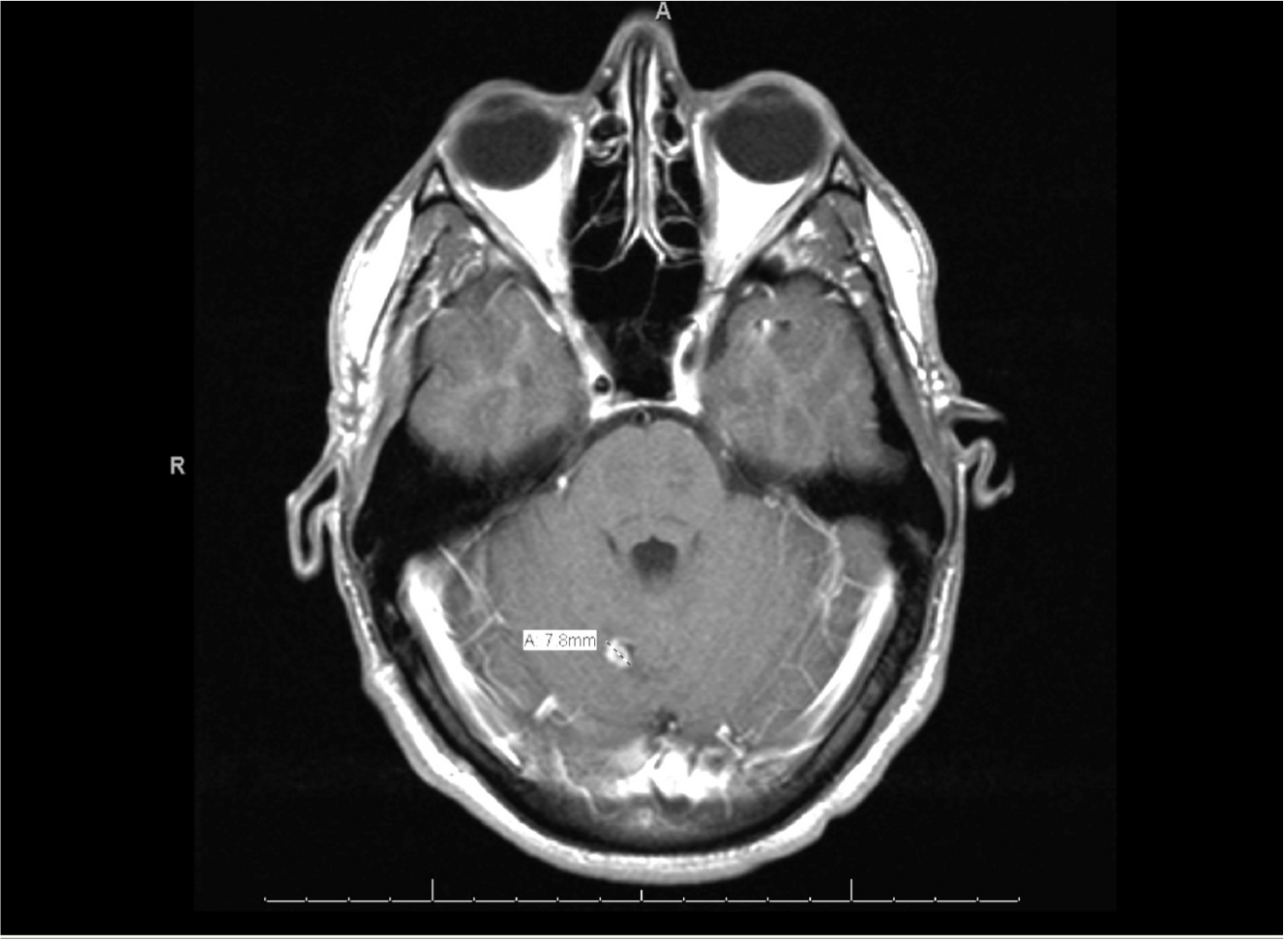
MRI One Month Post-Resection New definite enhancing foci compatible with metastatic foci.

Staging investigations with computed tomography (CT) and positron emission tomographic (PET) scans revealed pulmonary and pelvic tumor deposits. A scrotal ultrasound revealed a minimally atrophic right testicle with no further abnormalities detected. A follow-up cranial MRI revealed enhancement in the surgical bed and new metastatic foci (Figures 2, 3).

**Figure 4:**
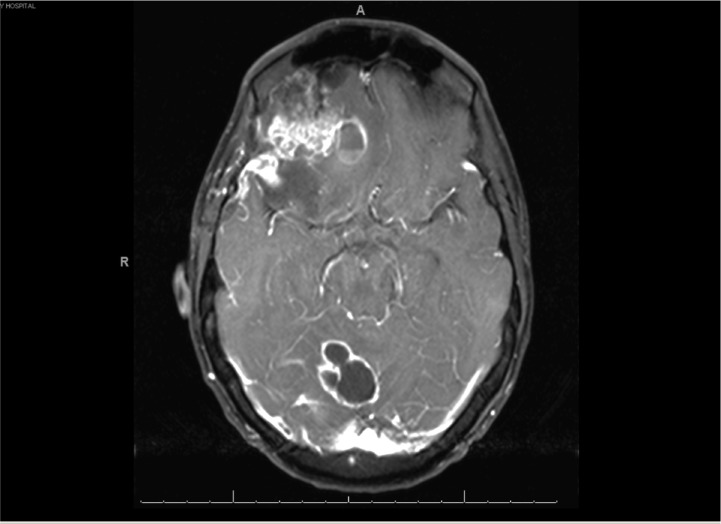
MRI Five Months After Initial Presentation Marked progression in a multiple ring-enhancing lesions with vasogenic edema.

Pathological and imaging findings were consistent with metastatic carcinoma with progressive brain lesions from an unestablished primary focus. At this time the brain lesions were increasingly symptomatic. Further treatment options with chemotherapy and whole brain radiation therapy (WBRT) were discussed with the patient. The patient refused palliative intent chemotherapeutic intervention for unknown primary, but agreed to WBRT with a prescribed dose of 30 gray in 10 fractions delivered. Subsequent to this, additional remote pathological consultation with chromosomal analysis revealed isochrome 12p amplications, consistent with a TGCT. Tumor markers with alpha-fetoprotein (aFP), beta-human chorionic gonadotropin (BhCG), and lactate dehydrogenase (LDH) were not elevated. With evidence supporting the potential for response to chemotherapeutic intervention in TGCT, the patient was started on cisplatin and etoposide, with the plan to include bleomycin in subsequent cycles if his pulmonary function improved [14-16]. Unfortunately the patient’s clinical course consisted of progressive brain metastases (Figure 4), seizures, and pulmonary embolism.

He rapidly deteriorated before receiving a full course of treatment and succumbed to his disease only five months after initial presentation. Informed consent was obtained from the patient initially and from the patient's family after he passed away.

## Discussion

### Literature review

English publications of ‘burned out’ TGCT case reports were identified from Medline and EMBASE databases via OVID engine without restrictions on year of publication. The keywords were “germ cell tumor,” “burned out phenomenon,” and “testicular tumor.” Additional studies were identified from reference lists of retrieved papers and review articles. Studies that did not discuss primary testicular origin were excluded. The search yielded 38 results and each abstract was reviewed. A total of 27 articles were thoroughly reviewed and 79 cases of ‘burned out’ TGCTs were identified. The presenting sites, age of patient, tumor markers, histology, treatments employed, and outcomes were tabulated (Table 1) [5, 7-8, 17-40]. 

**Table 1:** Reported Cases of ‘Burned Out’ TGCT n = number of cases aFP = alpha-fetoprotein BhCG = beta-human chorionic gonadotropin LDH = lactate dehydrogenase N = normal level + = elevated level BEP = bleomycin, etoposide, cisplatin RPLND = retroperitoneal lymph node dissection NR = not reported NOS = not otherwise specified

First Author/Citation	Year of Study	Presenting/Metastatic Site	Age of Patient	Tumor Markers		Histology		Treatment			Outcome		
Balalaa N [17]	2011	Retroperitoneal (n=1)	31	aFP	N	NR		BEP			Treatment response		
				BhCG	N								
				LDH	+								
Balzer BL [18]	2006	Retroperitoneal (n=20)	17-67 (mean 32)	BhCG	+ (n=2)	Seminoma (n=26)		NR			NR		
		Widely disseminated tumor (n=2)											
		Lung and Liver (n=1)											
		Mediastinum (n=1)											
		Other (thyroid, neck thoracic cavity) (n=1)											
		Testicular mass (n=7)											
Castillo C [19]	2003	Retroperitoneal (n=1)	25	aFP	+	Mature teratoma		BEP			Initial clinical response		
				BhCG	+								
Comiter CV [20]	1995	Retroperitoneal (n=1)	22-36	NR		NR		NR			NR		
		Supraclavicular (n=1)											
Curigliano G [21]	2006	Retroperitoneal (n=1)	42	aFP	N	Seminoma		Orchiectomy, BEP, and RPLND			NR		
				BhCG	+								
				LDH	N								
Fabre E [5]	2004	Testicular (n=1)	32	aFP	N	Seminoma		Orchiectomy and Radiotherapy (30Gy)			Free of disease 16 years after the diagnosis		
				BhCG	N								
				LDH	N								
		Retroperitoneal (n=1)	35	aFP	+	Mature teratoma		Orchiectomy, BEP plus vincristine, and RPLND			Free of disease 6 years after the diagnosis		
				BhCG	+								
		Retroperitoneal (n=1)	50	aFP	N	Seminoma		Orchiectomy, RPLND, and EP			Total remission 3 years after the diagnosis		
				BhCG	N								
				LDH	+								
		Retroperitoneal (n=1)	17	aFP	N	Mature teratoma		BEP, retroperitoneal mass resection, and orchiectomy			Free of disease 4 years after the diagnosis		
				BhCG	N								
				LDH	N								
		Supraclavicular (n=1)	39	aFP	N	Seminoma		BEP followed by salvage chemo (vinblastine, etoposide, ifosfamide, and cisplatin)			Total remission 3 years after the diagnosis		
				BhCG	N								
				LDH	N								
George SA [22]	2015	GIST (n=1)	24	aFP	N	Mixed GCT		Orchiectomy			NR		
				BhCG	N								
				LDH	N								
Gurioli A [23]	2013	Retroperitoneal (n=1)	35	aFP	N	Seminoma		BEP and orchiectomy			Free of disease 2 years after the diagnosis		
				BhCG	N								
				LDH	+								
		Retroperitoneal (n=1)	50	aF	N	Seminoma		Orchiectomy, vincristine, ifosfamide, bleomycin, and surgical debulking of mass			Free of disease 4 years after the diagnosis		
				BhCG	+								
Hu B [24]	2015	Retroperitoneal (n=1)	37	aFP	N	Seminoma		NR			NR		
				BhCG	N								
				LDH	N								
Jaber S [25]	2010	Retroperitoneal (n=1)	32	aFP	N	Seminoma		Orchiectomy and surgical removal of the retroperitoneal mass			NR		
				BhCG	N								
Kebapci M [26]	2001	Supraclavicular (n=1)	22	aFP	N	GCT having choriocarcinoma and probable embryonal cell carcinoma components		Orchiectomy and BEP			NR		
				BhCG	+								
Leleu O [27]	2000	Pulmonary (n=1)	30	aFP	+	Malignant germ cell tumor		Orchiectomy and BEP			Stable 3 years after the diagnosis		
				BhCG	+								
Lopez JI [28]	1994	Retroperitoneal (n=1)	20	aFP	N	Choriocarcinoma		Orchiectomy, biopsy of retroperitoneal masses, BEP plus vincristine			Deceased 7 months after initial complaints		
				BhCG	+								
				LDH	+								
Mesa H [29]	2009	Gastric ulcers (n=1)	55	aFP	N	Poorly differentiated adenocarcinoma- further studies revealed seminoma		Orchiectomy, vincristine, ifosfamide and cisplatin			Free of disease 1 years after the diagnosis		
				BhCG	N								
Onishi K [30]	2014	Para-neoplastic neurological syndrome (n=1)	41	NR		Seminoma		Orchiectomy and chemotherapy			Free of disease 15 months after the diagnosis		
Patel MD [8]	2007	Testicular (n=1)	23	aFP	N	Mixed GCT		Orchiectomy			NR		
				BhCG	N								
				LDH	N								
Perimenis P [31]	2005	Retroperitoneal (n=1)	40	aFP	N	Seminoma		Orchiectomy, resection of retroperitoneal mass, and radiotherapy to para-aortic nodes			Free of disease 2 years after the diagnosis		
				BhCG	N								
				LDH	N								
Peroux E [32]	2012	Retroperitoneal (n=1)	18	aFP	+	Non-seminoma NOS		Orchiectomy and chemotherapy			Full remission		
				BhCG	N								
Preda O [33]	2011	Retroperitoneal (n=1)	43	aFP	N	Seminoma		Orchiectomy and chemotherapy			Free of disease 5 months after the diagnosis		
				BhCG	N								
				LDH	+								
Qureshi JM [34]	2014	Retroperitoneal and Pulmonary masses (n=1)	20	aFP	N	Teratoma GCT		BEP followed by orchiectomy, RPLND, and hepatic mass resection			Free of disease 2 years after the diagnosis		
				BhCG	+								
				LDH	+								
Rzeszutko M [35]	2015	Spermatic cord (n=1)	56	aFP	N	Non-seminoma NOS		Resection of spermatic cord mass			Free of disease 6 months post operatively		
				BhCG	N								
Sahoo PK [36]	2013	Retroperitoneal (n=1)	33	aFP	N	Seminoma vs poorly differentiated carcinoma (seminoma confirmed on IHC)		Orchiectomy and BEP			Patient under observation at time of publication		
				BhCG	N								
				LDH	N								
Suzuki K [37]	1998	Mediastinum (n=1)	27	aFP	N	Teratoma GCT and sarcomatous elements		BEP			NR		
				BhCG	N								
Tasu J [7]	2003	Retroperitoneal (n=1)	23	NR		Non-seminoma NOS (n=3)	seminoma (n=2)	Non-seminoma NOS: BEP (n=3)	Metastatic seminoma: radiotherapy and RPLND (n=1)	Seminoma: orchiectomy (n=1)	Complete remission (n=3)	Free of disease after 5 year follow up (n=1)	Free of disease after 7 year follow up (n=1)
		Retroperitoneal (n=1)	35										
		Retroperitoneal (n=1)	50										
		Retroperitoneal (n=1)	17										
		Supraclavicular (n=1)	33										
Yamamoto H [38]	2007	Gastric tumor (n=1)	39	aFP	N	Seminoma		Orchiectomy and EP			Free of disease 2 years after the diagnosis		
				BhCG	N								
				LDH	+								
Yucel M [39]	2009	Retroperitoneal (n=1)	28	aFP	N	‘Burned out’ testicular tumor NOS		Orchiectomy and BEP			Free of disease 5 years after the diagnosis		
				BhCG	N								
				LDH	+								
Yucel M [40]	2009	Prostate (n=1)	49	aFP	N	Seminoma		Orchiectomy, BEP plus vincristine, and radiotherapy to mediastinum retroperitoneal and pelvic lymph nodes			Free of disease 7 years after the diagnosis		
				BhCG	N								

### Results

The sites of symptomatic metastasis identified were retroperitoneal (51.9%), testicular (12.7%), mediastinal (3.8%), pulmonary (3.8%), gastric (3.8%), and others (24.1%) consisting of prostate, supraclavicular, head and neck, and widely disseminated. The average patient age at presentation was 32.7 years old. Tumor markers were not found to be consistently elevated, with only 12.7%, 10.2%, and 5.1% of the cases found to be increased for BhCG, aFP, and LDH respectively. The most common treatment employed was orchiectomy with chemotherapy (57.5%), followed by chemotherapy alone (32.5%). Radiation therapy was utilized in four (10%) cases, all of which were seminoma [5,7,31,40]. The majority of reported cases had a good treatment response with only one reported death in the literature [28]. Tabulated case details are summarized in Table 2 [5, 7-8, 17-40].

**Table 2:** Summary of 'Burned Out' TGCT Cases NSGCT= non-seminomatous germ cell tumors +BhCG= elevated beta-human chorionic gonadotropin level +aFP= elevated alpha-fetoprotein level LDH= elevated lactate dehydrogenase level Orch= orchiectomy

Presenting Site of 'Burned Out' TGCT		Total Cases	Age (Mean, Range)	+BhCG	+aFP	+LDH	Orch Alone	Chemo Alone	Orch + Chemo	Radiation Therapy Included	Treatment Unknown	Treatment Response, Death, Outcome Unknown
Retroperitoneal		41	32 (17-67)					4	9	2	21	15,1,26
	*Seminoma*							4		2		
	*NSGCT*											
Testicular		10	33, (23-56)				2			1	7	2,0,8
	*Seminoma*									1		
	*NSGCT*						2					0,0.3
Mediastinum		3	30.3 (27-32)					1			2	
	*Seminoma*											
	*NSGCT*							1				
Pulmonary		3	27.3 (20-32)						2			2,0,1
	*Seminoma*											
	*NSGCT*								2			
Gastric		3	39 (24-55)					1	2			
	*Seminoma*								2			
	*NSGCT*											
Other		19	32 (20-49)					1	3	1	8	4,0,13
	*Seminoma*								1	1		
	*NSGCT*							1	2			
Total		79	32.7 (17-67)	10	4	8	4	13	16	4	38	23,1,51
	*Seminoma*				0	4				4		
	*NSGCT*				4	2						
	*Other/unknown*					2						

### Case discussion

A salient feature of all invasive TGCTs is a gain in material in the short arm of chromosome 12, and is diagnostic if present [41]. Although the initial pathology revealed a non-diagnostic metastatic tumor, further testing revealed an amplification of chromosome 12p leading to the diagnosis of TGCT. This suggests that the examination of poorly differentiated carcinomas of an unknown primary site using light microscopy and immunohistochemical profiling may be inadequate, and should undergo additional testing modalities with molecular chromosomal analysis [41-42].

The behavior and aggressive nature of the tumor discussed throughout this case combines the complexity of the evolving field of tumor biology and unique patient characteristics. Interestingly, the patient had a confirmed family history of factor V Leiden mutation. It has been suggested that clotting factor polymorphisms such as factor V Leiden are associated with cancer onset and progression. The theoretical mechanism behind such adverse effects stems from the involvement of tissue factor and thrombin in tumor angiogenesis, which is essential for tumor growth and metastasis [43]. Furthermore, such factors may contribute to a more radio-resistant tumor profile despite advanced diagnostic techniques and treatment modalities. Thus, this case reflects the arising need for further research to explore the dynamic interplay of tumor biology and patient characteristics for targeting tumor response.

## Conclusions

This case is presented for its unconventional presentation, rarity of occurrence, and difficulty in diagnosis. It brings forward the discussion of both the commonality of TGCT in young male adults, as well as the anomaly of a ‘burned out’ TGCT. With unreliable tumor markers, nonspecific symptoms, and pathological findings, the ‘burned out’ phenomenon accounts for a challenging diagnosis, particularly with the presenting symptom arising from a less common metastatic site. This case adds to the increasing literature on the rare entity of the ‘burned out’ TGCT, and upon literature review, presents itself as the first reported case presenting with brain metastasis. By establishing a strong foundation of ‘burned out’ TGCT in the literature leading to familiarity of the diagnostic process, a deeper understanding into medical management may arise.
